# Micro‐histology combined with cytology improves the diagnostic accuracy of endometrial lesions

**DOI:** 10.1002/cam4.6338

**Published:** 2023-07-17

**Authors:** Yiran Wang, Lanbo Zhao, Kailu Zhang, Yu Liu, Lin Guo, Wei Jing, Huilian Hou, Guizhi Shi, Yadi Bin, Siyi Zhang, Guanjun Zhang, Qiling Li

**Affiliations:** ^1^ Department of Obstetrics and Gynecology The First Affiliated Hospital of Xi'an Jiaotong University Xi'an Shaanxi China; ^2^ Department of Pathology The First Affiliated Hospital of Xi'an Jiaotong University Xi'an Shaanxi China; ^3^ Aviation General Hospital of Beijing Medical University and Beijing Institute of Translational Medicine, University of Chinese Academy of Sciences Beijing China

**Keywords:** CB, cytology, endometrial cancer, LBC, micro‐histology

## Abstract

**Background:**

In the study, we aimed to evaluate the ability of micro‐histology combined with cytology to improve the quality of slides and diagnose endometrial lesions.

**Methods:**

Endometrial specimens were collected from Li Brushes. Every specimen was prepared for micro‐histological and cytological slides, using cell block (CB) and liquid‐based cytology (LBC) technologies. Semi‐quantitative scoring system was used to evaluate the qualities of slides. CB slides were assessed by 5‐category scoring system. Diagnostic accuracy was calculated in LBC, CB, and LBC + CB groups based on the histological gold standard. Endometrial atypical hyperplasia, and endometrial cancer were considered positive, whereas others were considered negative.

**Results:**

A total of 167 patients were enrolled. CB slides were inferior to LBC slides only in cellularity (*p* < 0.001), but superior in the other six parameters (all *p* < 0.001). The satisfaction rate of micro‐histology accounted for 92.3%. The accuracy index in the CB group was higher than in the LBC group in terms of sensitivity (85.5% vs. 82.7%) and specificity (98.9% vs. 95.7%). The sensitivity and specificity in the LBC + CB group were increased to 94.2% and 99.0%, respectively.

**Conclusions:**

The quality of micro‐histological slides was higher than that of cytological slides. By combining micro‐histology with cytology, higher accuracy was achieved for endometrial lesions diagnosis.

## 1 INTRODUCTION

Endometrial cancer (EC) is the most common malignant tumor of female reproductive system with 66,200 newly diagnosed patients in the United States in 2023.^1^ The International Federation of Gynecology and Obstetrics stage reflects the prognosis closely, in which the 5‐year survival of Stage I is 85% and Stage IV EC is only 25%.^2^ Additionally, patients with advanced EC require lymphadenectomy, which greatly affects their quality of life.^3^ Thus, early detection of EC or atypical endometrial hyperplasia is crucial. Dilatation and curettage (D&C) and hysteroscopic excisional biopsy are regarded as the gold standards for evaluating endometrial lesions and prognosis^4^; however, their invasiveness limits their wide usage in EC screening. Office biopsy has been recommended as the method of choice for histological evaluation of the endometrium in America,^5^ Britain,^6^ and Europe^7^ because it is minimally invasive, reliable, and accurate. Endometrial cytology test (ECT) is another effective, painless, and office‐sampled diagnostic approach. It was established in the *Japan Health Insurance Law* in 1987,^8^ and is listed in German^9^ and Chinese guidelines.^10^ However, it is not easy to differentiate malignancies from benign lesions based on cytology alone, because endometrial cells experience periodic morphological changes accompanied by menstrual cycle.^11^ Besides, excess blood, mucus, and overlapping cells make a precise diagnosis using liquid‐based cytology (LBC) slides difficult.^12^ Some microtissues are observed in specimens collected using brushes.^13^ However, these are usually discarded during the preparation of traditional LBC slides. The cell block (CB) technique is used to collect sediment and microtissue from cytological specimens. After being processed into a solid block using various methods, the microtissues are embedded in paraffin, cut and stained similarly to histological slides.^14^ The histological‐like processing often strengthens architectural features and relationships between distinct cell populations,^15^ rendering it easy to diagnose.

Our team has invented a gel fixative called a diluted solution (patent num. ZL. 2017.2.0297254.3, Xi'an Meijiajia Medical Technology Co., Ltd.) to prepare CB slides. In this study, CB and LBC slides were prepared for each specimen, and we evaluated whether micro‐histology could improve the quality of the slides and the diagnostic accuracy of endometrial lesions compared to cytology.

## 2 MATERIALS AND METHODS

### 2.1 Patients

Patients enrolled in this diagnostic study were those who underwent D&C with hysteroscopy in outpatient clinic or hysterectomy in inpatient department of the First Affiliated Hospital of Xi'an Jiaotong University between June 2019 and June 2021. Patients who underwent hysterectomy for cervical cancer, cervical intraepithelial neoplasia, or ovarian cancer were excluded.

### 2.2 Sampling

Endometrial specimens were collected using Li Brushes (Xi'an Meijiajia Medical Technology Co., Ltd.) prior to D&C or hysterectomy. First, a gynecological examination was performed, the depth of the uterine cavity was measured, and a sheathed Li Brush was gently inserted to the level of the uterine fundus. Second, the outer sheath was pulled back by ~5 mm to expose the bristle, and the brush was rotated 5–10 times. Third, the outer sheath was advanced by 3 mm to erect the bristle, and the brush was rotated for 5–10 circles to gather cells and microtissues from the uterine fundus. Finally, Li Brush was retracted into its outer sheath prior to withdrawal through the uterus.^16^ The bristle was then immersed in an endometrial preservation solution^17^ (Xi'an Meijiajia Medical Technology Co., Ltd.), and rotated to release cells and microtissues.

### 2.3 Preparation of pathological slides

After sampling, 3–5 mL erythrocyte lysate was added to remove blood, and 5 mL of the specimen was transferred into a sample chamber (Figure S1A), and placed in the Li‐Shi Thin Prep Liquid‐based Cytology and Tissue Embedding Machine (shorted as Li‐Shi Machine) (Xi'an Meijiajia Medical Technology Co., Ltd.) (Figure S1B). After centrifugation at 1500 rpm for 10 min using “Cell Centrifugation” pattern, cells were transferred to a slide (Figure S1C) and then stained with Papanicolaou method (Figure S1D). These slides were enrolled in the LBC group.

The residual specimens were processed into CB slides, designated as the CB group. The diluted solution, which was solid at room temperature, was melted at 80°C in a water bath (Figure S2A). Then, the residual specimens were poured into 15 mL centrifuge tubes and placed into Li‐Shi Machine (Figure S2B). “Cell Centrifugation” pattern was chosen at 2000 rpm for 10 min (Figure S2C). After centrifugation, cells and microtissues were deposited at the bottom of centrifuge tubes (Figure S2D). Supernatant was discarded, and sediment was transferred into a melted diluted solution (Figure S2E). Subsequently, specimens were loaded into Li‐Shi Machine again (Figure S2F), and polymerized at 2000 rpm for 10 min using “Cell Polymerization” pattern (Figure S2G). After being cooled to solid at room temperature (or in a refrigerated room) (Figure S2H), the part without a cell‐rich layer was cut off. The specimens were embedded in paraffin wax using the automatic embedding machine, and 3 μm slides were stained with hematoxylin and eosin (HE) (Figure S2I).

The histological specimens from D&C or hysterectomy were routinely fixed in neutral‐buffered formalin, embedded in paraffin, and stained with HE in the Pathology Departments.

### 2.4 Evaluation method

The quality of LBC and CB slides was evaluated using semi‐quantitative scoring system (Table 1)^17^ including the following seven parameters: cellularity, background blood debris, informative background, monolayer, cell architecture, nuclear, and cytoplasmic details.

**TABLE 1:** Semiquantitative scoring system of cytological feature.

Cytological feature				
Cellularity	Zero	Scanty	Adequate	Abundant
Background blood debris	Zero	Occasional	Good amount	Abundant
Informative background	Absent	Present	—	—
Monolayer	Absent	Occasional	Good amount	—
Cell architecture	Non‐recognized	Moderately recognized	Well recognized	—
Nuclear details	Poor	Fair	Good	Excellent
Cytoplasmic details	Poor	Fair	Good	Excellent

For CB slides, the 5‐category score system was used to assess the quantities and qualities of endometrial glands^18^: Category 1: only mucus or bleeding components; Category 2: a small amount of superficial mucosal epithelial components; Category 3: visible endometrial glands (<5); Category 4: visible endometrial glands^5^, ^6^, ^7^, ^8^, ^9^, ^10^; and Category 5: visible endometrial glands (≥ 10).

Histological diagnoses were performed by colleagues of the Pathology Departments following World Health Organization 2020 criteria (Table 2)^19^; the results were recognized as the gold standard. Based on the endometrial micro‐histological criteria proposed by Chinese Expert Consensus^10^ and cytological diagnostic criteria reviewed by Maksem JA et al.,^20^ LBC and CB slides were blindly reviewed by two experienced pathologists (HH and GS, with over 20 years of endometrial experience). Discussion were held between two professors if different diagnoses were made. If the discussion failed to conclude an accurate and consistent diagnosis, a third pathologist (GZ) made the final diagnosis. Slides were considered invalid and excluded from the diagnostic test when cells or microtissues were too small for diagnosis. The validation rate referred to the proportion of valid slides to the total number of slides.

**TABLE 2:** Characteristics of patients enrolled in the group.

Characteristics	*N*
Source	
Outpatient patient	83
Hospitalized patient	84
Age	
<40	20
≥40	147
Clinical diagnosis	
AUB‐O	72
Uterine leiomyoma	24
EC	51
Adenomyosis	11
Endometrial polyps	4
Uterine prolapse	2
Endometrial atypical hyperplasia	2
Thickened endometrium	1
Pathological diagnosis	
Proliferative endometrium	22
Secretory endometrium	14
Atrophic endometrium	14
Mixed endometrium	1
Endometrial hyperplasia without atypia	56
Endometrial atypical hyperplasia	2
Endometrial carcinoma	51
Endometrioid carcinoma, G1/G2	42
Endometrioid carcinoma, G3	6
Serous carcinoma	1
Clear cell carcinoma	1
Not available	7

Abbreviations: AUB‐O, abnormal uterine bleeding‐ovulation; EC, endometrial cancer.

The results of diagnostic tests in LBC, CB, and LBC + CB groups were compared with those of histopathology obtained after D&C or hysterectomy (the gold standard). Positive results were defined as EC and endometrial atypical hyperplasia, normal endometrium, and endometrial hyperplasia without atypia were considered negative. In LBC + CB group, one or more positives were considered positive, and all negatives were considered negative.

### 2.5 Statistical analysis

The “Diagnostic Tests—Tests for One‐Sample Sensitivity and Specificity” of PASS 11.0.4 software was used to calculate sample size. The predicted sensitivity (Se) and specificity (Sp) were set to 89.7% and 97.4%, respectively,^21^ *α* = 0.05, *β* = 0.1.

Paired four lattices were created to appraise the accuracy of LBC, CB, and LBC + CB groups respectively, including Se, Sp, false‐negative rate (FNR), false‐positive rate (FPR), positive likelihood ratio (LR+), negative likelihood ratio (LR−), positive predictive value (PV+), negative predictive value (PV−), and Youden index (*r* = 1 − (FNR + FPR)). These indices and their corresponding confidence intervals (CIs) were calculated using http://vassarstats.net/clin1.html.

The comparison of validation rates was performed using the chi‐squared test using the SPSS software (version 10.0). The scores for the seven cytological features in LBC and CB groups were compared using paired *t*‐tests. Statistical significant difference was defined as *p* < 0.05.

## 3 RESULTS

### 3.1 Patients

After calculation, at least 45 positive and 63 negative specimens were required. Finally, total 167 patients participated in our study, including 53 positive, 107 negative, and 7 invalid D&C cases. Among them, 83 (49.7%) were outpatient patients, and 84 (50.3%) were hospitalized patients. The ages of patients ranged from 23 to 81 years, and the median age was 49 years. There were 20 patients under 40 years and 147 patients over 40 years old. The clinical diagnoses included: abnormal uterine bleeding‐ovulation (AUB‐O) (72, 43.1%), EC (51, 30.5%), uterine leiomyoma (24, 14.4%), adenomyosis (11, 6.6%), endometrial polyps (4, 2.4%), uterine prolapse (2, 1.2%), endometrial atypical hyperplasia (2, 1.2%), and thickened endometrium (1, 0.6%). The pathological diagnoses were: proliferative endometrium (22, 13.2%), secretory endometrium (14, 8.4%), atrophic endometrium (14, 8.4%), mixed endometrium (1, 0.1%), endometrial hyperplasia without atypia (56, 33.5%), endometrial atypical hyperplasia (2, 1.2%), endometrioid carcinoma, G1/G2 (42, 25.1%), endometrioid carcinoma, G3 (6, 3.6%), serous carcinoma (1, 0.6%), clear cell carcinoma (1, 0.6%), and mixed carcinomas (1, 0.6%). In 7 cases (4.2%), the histological specimens collected by D&C were insufficient to be diagnosed (Table 2).

### 3.2 Pathologic images

**FIGURE 1:**
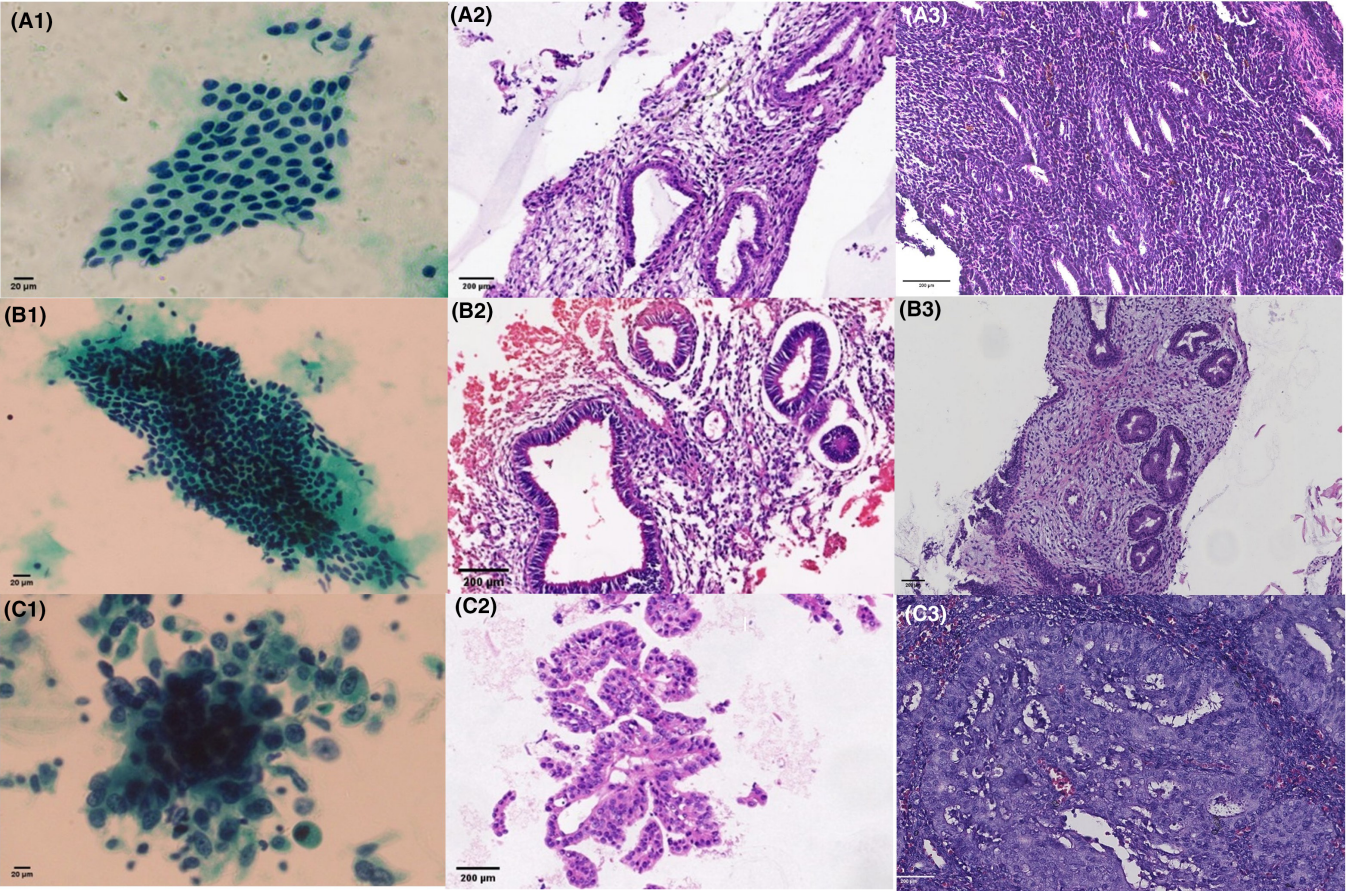
Cytological (Papanicolaou), micro‐histological (hematoxylin and eosin), and histological images (hematoxylin and eosin). (A) Normal endometrium. (B) Endometrial hyperplasia without atypia. (C) EC.

After scanning LBC slides with a microscope camera, we obtained different types of cytopathological images: normal endometrial cells (Figure 1A1), endometrial hyperplasia cells without atypia (Figure 1B1), and EC cells (Figure 1C1). Similarly, the corresponding micro‐histological images of CB slides were obtained: normal endometrial (Figure 1A2), endometrial hyperplasia without atypia (Figure 1B2), and EC (Figure 1C2). Corresponding histological images were shown in Figure 1A3–C3.

### 3.3 Validation rate

Among the 167 LBC slides, 8 with insufficient cellularity were diagnosed as invalid, including 1 D&C failed case, 6 histologically negative cases, and 1 positive case. Eleven of the 167 CB slides were invalid, with 1 failed D&C case, 8 negative, and 2 positive cases histologically. Among the 83 D&C cases, 7 were invalid specimens, 6 were diagnosed as benign lesions by LBC and CB, and 1 case was invalid for both LBC and CB. Only 1 case failed for all methods. In this study, the specimen validation rate did not show statistically significant difference among LBC (95.2%), CB (93.4%), and D&C groups (91.6%) (*χ* ^2^ = 1.318, *p* = 0.517).

### 3.4 Cytological satisfaction

According to the results of paired *t*‐test, statistically significant differences were observed between the satisfaction scores of CB and LBC groups regarding cellularity (1.61 ± 0.66 vs. 2.30 ± 0.84, *p* < 0.001), background blood debris (1.17 ± 0.80 vs. 1.95 ± 1.12, *p* < 0.001), informative background (0.82 ± 0.40 vs. 0.62 ± 0.49, *p* < 0.001), monolayer (1.51 ± 0.62 vs. 1.15 ± 0.75, *p* < 0.001), cell architecture (1.59 ± 0.59 vs. 1.20 ± 0.69, *p* < 0.001), nuclear details (1.42 ± 0.74 vs. 0.48 ± 0.55, *p* < 0.001), and cytoplasmic details (1.41 ± 0.73 vs. 0.44 ± 0.55, *p* < 0.001).

### 3.5 Micro‐histological satisfaction

**FIGURE 2:**
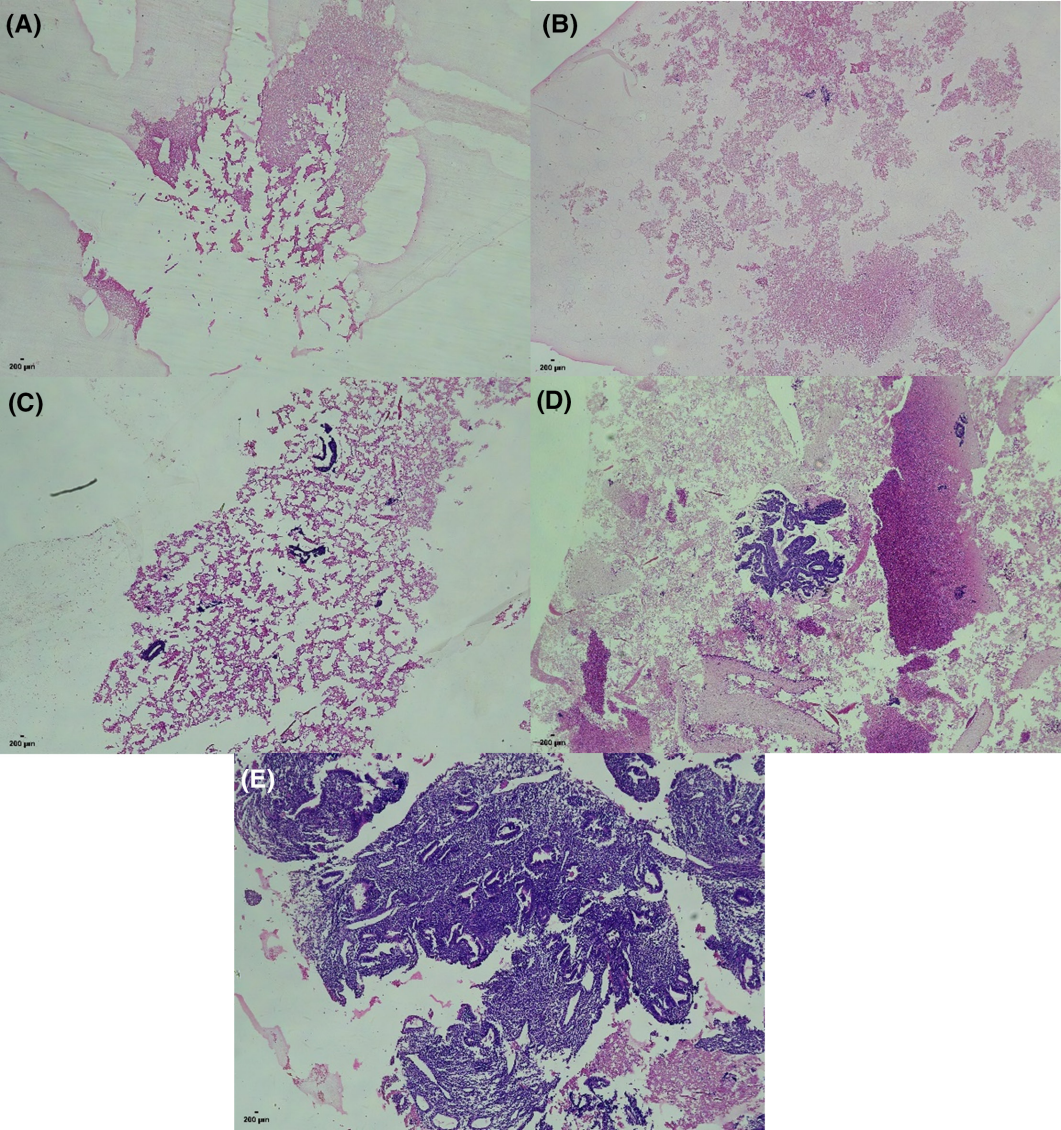
Different levels of satisfaction of CB slides (hematoxylin and eosin). (A) Category 1: only mucus or bleeding components. (B) Category 2: a small amount of superficial mucosal epithelial components. (C) Category 3: visible endometrial glands (<5). (D) Category 4: visible endometrial glands (5–10). (E) Category 5: visible endometrial glands (≥10).

Among the 167 CB slides, 11 belonged to slides of Category 1, 2 to Category 2, 8 to Category 3, 59 to Category 4, and 87 to Category 5 (Figure 2). The proportions of Category 4–5 and Category 3–5 were 87.4% and 92.2%, respectively.

### 3.6 Diagnostic accuracy calculation

**FIGURE 3:**
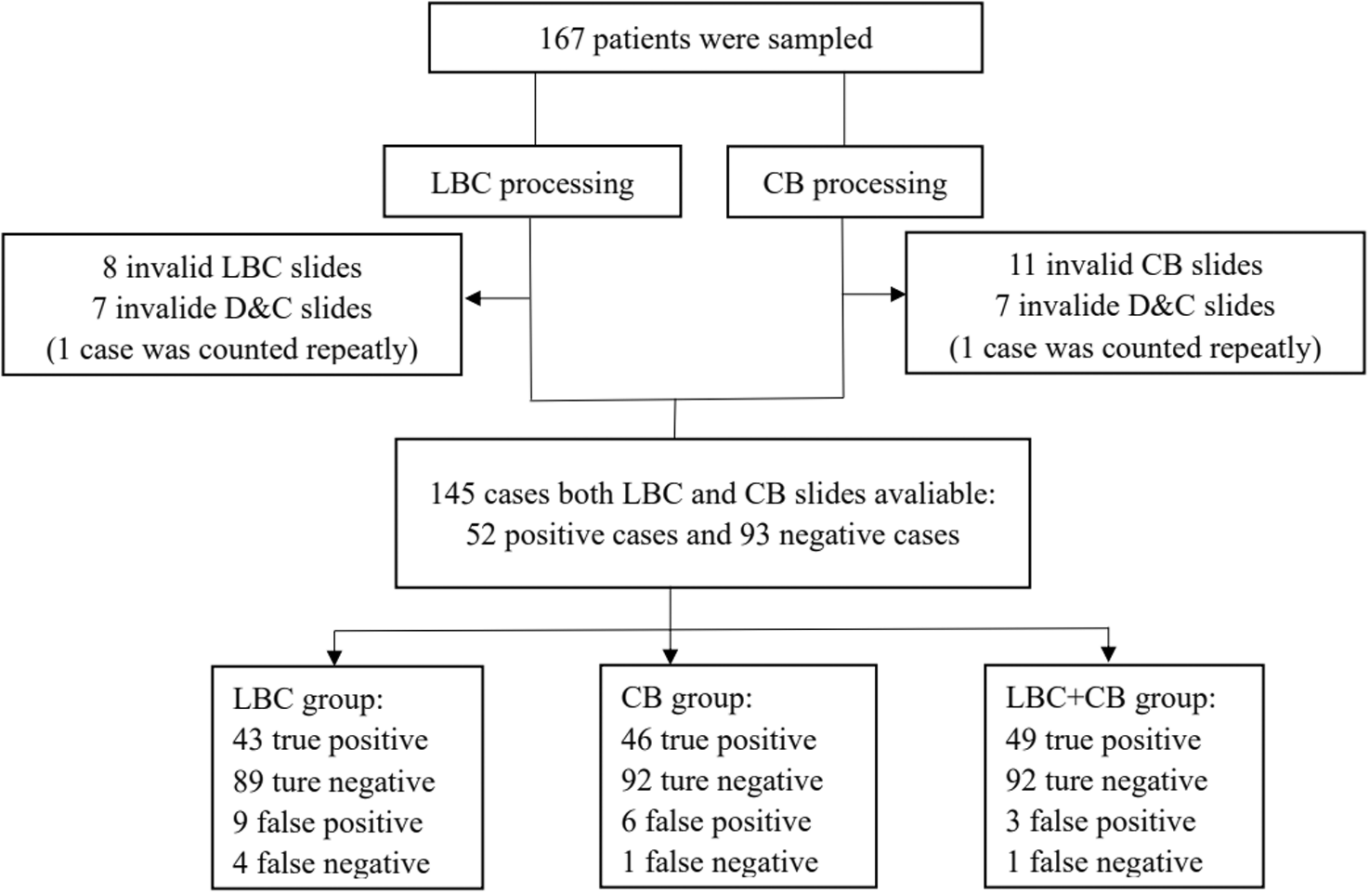
Diagram of study participants.

After removing invalid cases regardless of histological or micro‐histological/cytological sampling failure, 145 valid cases were incorporated into the diagnostic test (Table 3, Figure 3). There were 43 true‐positive, 89 true‐negative, 9 false‐positive, and 4 false‐negative cases in LBC group. Using CB slides alone, we correctly diagnosed 46 positive and 92 negative cases, 3 positive cases and 1 negative case improperly. There were 49 true‐positive, 92 true‐negative, 3 false‐positive, and 1 false‐negative cases in the LBC + CB group. After statistical calculations, the Se of LBC group was 82.7% (69.2%, 91.3%), and the Sp was 95.7% (88.7%, 98.6%). In CB group, Se was 88.5% (75.9%, 95.2%) and Sp was 98.9% (93.3%, 100.0%). Se increased to 94.2% (83.0%, 98.5%) in LBC + CB group, and Sp was 99.0% (93.3%, 99.9%). Other accuracy indices were listed in Table 4.

**TABLE 3:** Frequency of cases in LBC, CB, and LBC + CB group.

Method		Gold standard		Total
		+	−	
LBC	+	43	4	47
	−	9	89	98
	Total	52	93	145
CB	+	46	1	47
	−	6	92	98
	Total	52	93	145
LBC + CB	+	49	1	50
	−	3	92	95
	Total	52	93	145

Abbreviations: CB, cell block; LBC, liquid‐based cytology.

**TABLE 4:** Accuracy indices of LBC, CB, and LBC + CB group.

	LBC	CB	LBC + CB
Se (95% CI)%	82.7 (69.2, 91.3)	88.5 (75.9, 95.2)	94.2 (83.0, 98.5)
Sp (95% CI)%	95.7 (88.7, 98.6)	98.9 (93.3, 100.0)	99.0 (93.3, 99.9)
FNR (95% CI)%	17.3 (8.7, 30.8)	11.5 (4.8, 24.1)	5.8 (1.5, 17.0)
FPR (95% CI)%	4.3 (1.4, 11.3)	1.1 (0.0, 6.7)	1.0 (0.1, 6.7)
PV+ (95% CI)%	91.5 (78.7, 97.2)	97.9 (87.3, 99.9)	98.0 (88.0, 99.9)
PV− (95% CI)%	90.8 (82.8, 95.5)	93.9 (86.6, 97.5)	96.8 (90.4, 99.2)
LR+ (95% CI)	19.2 (7.3, 50.6)	82.7 (11.7, 579.3)	87.6 (12.5, 616.3)
LR− (95% CI)	0.18 (0.10, 0.33)	0.12 (0.05, 0.25)	0.06 (0.02, 0.17)
*r*	0.78 (0.58, 0.90)	0.87 (0.69, 0.95)	0.93 (0.76, 0.98)

Abbreviations: CB, cell block; FNR, false‐negative rate; FPR, false‐positive rate; LBC, liquid‐based cytology; LR−, negative likelihood ratio; LR+, positive likelihood ratio; PV−, negative predictive value; PV+, positive predictive value; *r*, Youden index; Se, sensitivity; Sp, specificity.

## 4 DISCUSSION

Early detection of EC is urgently needed for clinical applications; however, there are no undisputed methods worldwide. Researchers have conducted various studies seeking simple, painless, and convenient approaches. Some scholars have focused on blood biomarkers, such as the telomere length in circulating cell‐free DNA,^22^ lipid biomarkers,^23^ and microRNAs.^24^ Some researchers have found that Papanicolaou classifications,^25^ proteomics,^26^ and mutational analysis^27^ of cervical cell samples could be utilized to detect EC, and the presence of glandular cells was often associated with a poor prognosis.^28^ Some researchers have shown that a urine‐based proteomic profiling model is a potential tool for EC detection.^29^ However, these methods require further validation before they can be clinically used ECT has been widely used in several countries, and Osaki Study Group proposed a cytological diagnostic criteria to improve diagnosis^30^; however, it is still difficult to make a correct cytological diagnosis. Thus, we attempted to prepare additional micro‐histological slides using cytological samples, and combined them with cytological slides to reduce the difficulty of diagnosis.

Most fixatives used in the preparation of CB, except for mercuric chloride (HgCl_2_)‐based fixatives, did not offer an optimal morphological characterization of cells compared with corresponding smears,^31^ resulting in dissatisfaction with CB quality from cytopathologists.^32^ Moreover, HgCl_2_‐based fixative is potentially toxic, dangerous to laboratory personnel, and problematic for disposal.^33^ Formalin‐fixed tissues from surgical specimens have perfect histological structures.^34^ However, formalin showed the least satisfactory nuclear details when CB was fixed.^35^ Therefore, we developed a new fixative: a diluted solution; it is composed of compound materials with better elasticity and toughness than homogenous materials. The diluted solution becomes a melted gel when heated above 80°C, and becomes solid at room temperature. Rapid coagulation minimizes cell and microtissue losses. Therefore, theoretically, CB slides have high validity. Our results confirmed this conjecture, as the validation rate of CB with the diluted solution (93.4%) was significantly higher than that of formalin CB (77.8%)^21^ (*χ* ^2^ = 17.3, *p* < 0.001). Additionally, the cell architecture, nuclear details, and cytoplasmic details of CB were clearer than those of LBC. CB slides had less background blood debris and overlapping cells, and had a more abundant informative background than LBC slides. The main components of the diluted solution are gel, pure water and sodium chloride, which are harmless to the human body and easy to store. Thus, the use of a diluted solution is a breakthrough for CB, which will accelerate its application in various fields. In our study, a few LBC or CB slides were invalid because of insufficient cells or microtissues. Many factors such as age, menopausal status, body mass index, and endometrial thickness result in nondiagnostic specimens.^36^ Despite being the gold standard for diagnosing endometrial lesions, D&C cannot guarantee sufficient specimens every time as well,^37^ and the seven failed D&C cases in the current study also proved the fact. No statistically significant difference existed in the validation rates of LBC, CB, and D&C groups, suggesting that LBC and CB could be applied to diagnose endometrial lesions similar to D&C. Excluding one invalid D&C case, 85.7% (6 out of 7) LBC and 80% (8 out of 10) CB invalid ones were negative, resulting in little effect on the missed diagnoses of EC or endometrial atypical hyperplasia.

Many studies have shown that additional CB preparation from the residual LBC material is an effective method for improving the diagnostic accuracy of various diseases. Hui Zhang et al. reported 79.3% and 82.8% Se, 97.4% and 98.3% Sp in EC screening using LBC and CB separately, respectively. Moreover, LBC + CB demonstrated improved Se (89.7%) and Sp (97.4%).^21^ However, the invalidity rate of CB (22.2%) was significantly higher than for LBC (7.1%) (P < 0.01) in their study. In our study, there was no statistically significant difference between LBC and CB in validation rate, as previously explained. Our results were better than those of the above study, with a Se of 82.7%, 88.5%, and 94.2% and Sp of 95.7%, 98.9%, and 99.0% in LBC, CB, and LBC + CB groups, respectively. LBC + CB increased the detection rate of positive cases by 11.5% and 5.8% compared with LBC and CB alone, respectively, thereby reducing missed diagnosis rates.

Nevertheless, this study has several limitations. First, high‐risk groups requiring screening for EC are the target populations for ECT; however, we recruited symptomatic patients instead of asymptomatic populations in the study. Whether micro‐histology, combined with cytology, can detect EC in high‐risk populations requires further investigation. Second, we included 72 patients with AUB in the study but only performed additional cytological sampling, which did not interfere with the D&C procedure. In conventional clinical procedures, patients with heavy bleeding are not suitable for ECT because bleeding does not stop, unlike in D&C does. Third, though we enrolled more patients than calculated, this study had a small sample size, and more patients need to be included for further verification.

In conclusion, the quality of the micro‐histological slides was higher than that of the cytological slides. Considering the synergistic effects, micro‐histology can be integrated with cytology in clinical practice to improve the diagnostic accuracy of endometrial lesions.

## AUTHOR CONTRIBUTIONS

**Yiran Wang:** Writing – original draft (lead). **Lanbo Zhao:** Writing – review and editing (equal). **Kailu Zhang:** Resources (equal). **Yu Liu:** Methodology (equal). **Lin Guo:** Resources (equal). **Wei Jing:** Resources (equal). **Huilian Hou:** Methodology (equal). **Guizhi Shi:** Methodology (equal). **Yadi Bin:** Writing – review and editing (equal). **Siyi Zhang:** Writing – review and editing (equal). **Guanjun Zhang:** Conceptualization (equal); supervision (equal). **Qiling Li:** Conceptualization (lead); funding acquisition (lead).

## FUNDING INFORMATION

This work was supported by the Natural Science Basic Research Program of Shaanxi (2017ZDJC‐11, 2018JM7073), the Clinical Research Award of the First Affiliated Hospital of Xi'an Jiaotong University, China (XJTU1AF‐2018‐017, XJTU1AF‐CRF‐2019‐002), the Key Research and Development Program of Shaanxi (2017ZDXM‐SF‐068, 2019QYPY‐138), the Innovation Capability Support Program of Shaanxi (2017XT‐026, 2018XT‐002), the Medical Research Project of Xi'an Social Development Guidance Plan (2017117SF/YX011‐3), and the Bethune·Young and Middle‐aged Doctors Talent Training Program‐Women's Health Research Project (HX202046). The funders had no role in study design, data collection and analysis, decision to publish, or preparation of the manuscript.

## CONFLICT OF INTEREST STATEMENT

The authors declare no conflict of interests.

## ETHICS APPROVAL STATEMENT

This study was conducted in accordance with the Declaration of Helsinki, with the approval of Ethics Committee of the First Affiliated Hospital of Xi'an Jiaotong University (XJTU1AF2017LSK‐100).

## PATIENT CONSENT STATEMENT

All patients provided written informed consents after being sufficiently informed of the content of the study.

## PERMISSION TO REPRODUCE MATERIAL FROM OTHER SOURCES

Not available.

## CLINICAL TRIAL REGISTRATION

Clinical Trial Registration: http://www.chictr.org.cn, identifier ChiCTR1800020123.

## Supporting information


Figure S1‐S3

## Data Availability

The data that support the findings of this study are available from the corresponding author upon reasonable request.
