# Supplementary material for: Micro‐histology combined with cytology improves the diagnostic accuracy of endometrial lesions
Source: Cancer Med. 2023 Jul 17;12(16):17028–36. doi: 10.1002/cam4.6338 (PMC10501300; doi:10.1002/cam4.6338)
Supplement: Supplementary file 1 — Figure S1‐S3 [file CAM4-12-17028-s001.docx]

Supplementary Material


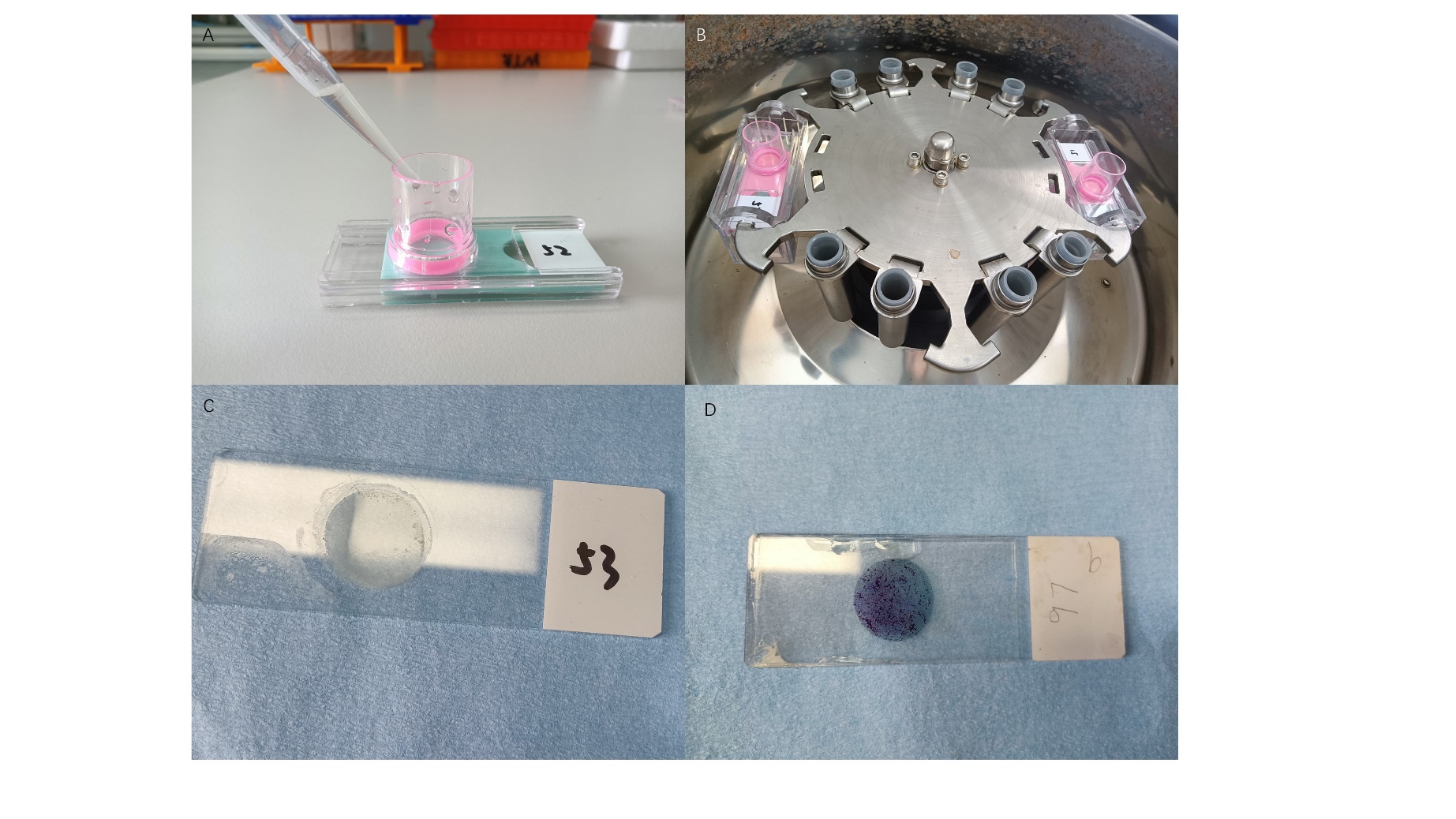


Sfig.1 **A** 5 ml specimen was transfered into the sample chamber; **B** Sample chambers were put in Li-Shi Machine; **C** Cells were transferred to the slide; **D** Slide with Papanicolaou staining.


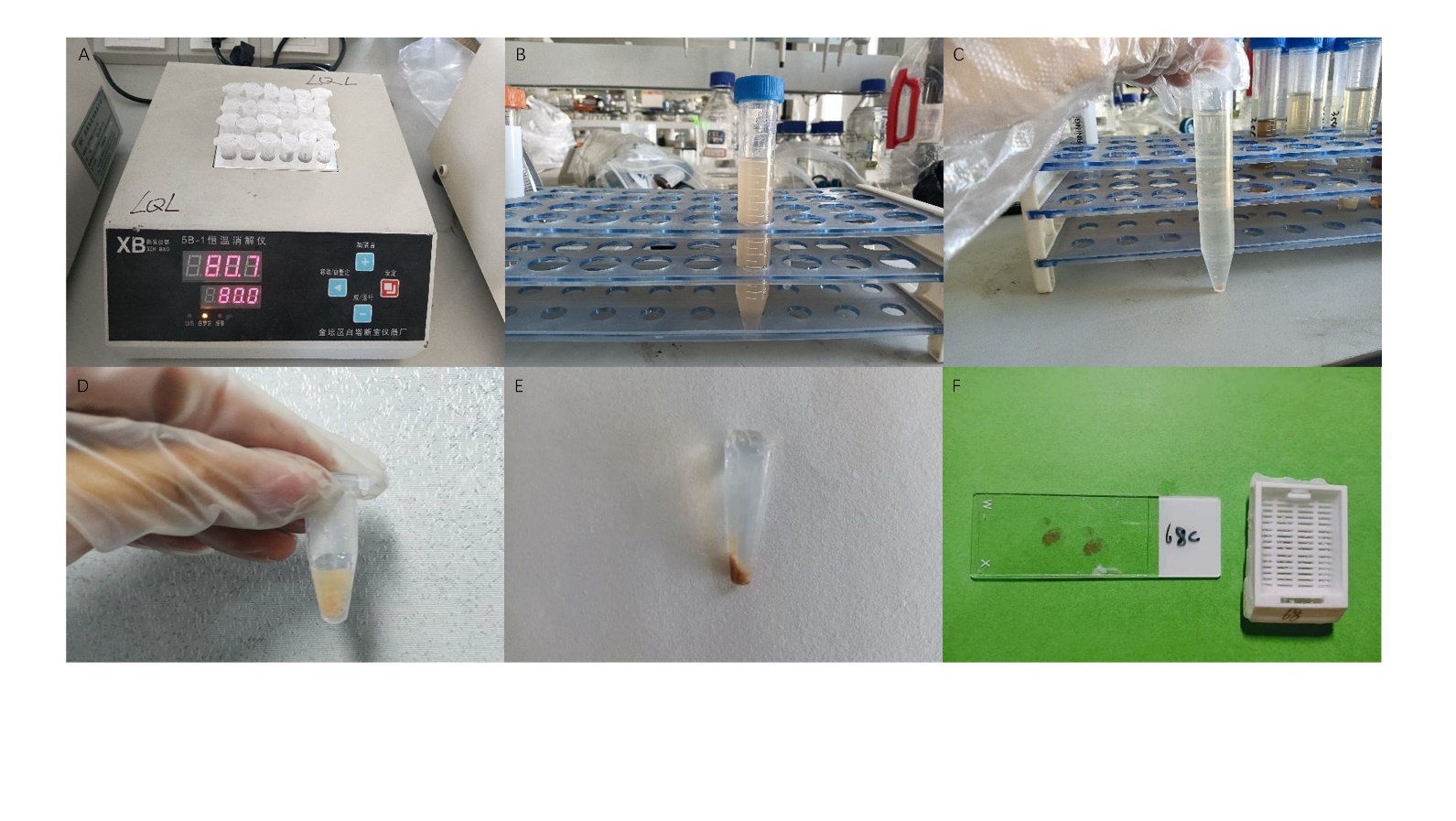


**Sfig.2 A** Diluted solution was melted at 80°C; **B** Residual specimen was put into 15ml centrifuge tube; **C** After centrifugation, cells and microtissues were deposited at the bottom of centrifuge tube; **D** Sediment was transferred into melted diluted solution; **E** Cell block was solidified into a solid; **F** Slides with hematoxylin and eosin staining.
